# Evaluating the forehead temperature for estimating the postmortem interval

**DOI:** 10.1007/s00414-026-03736-x

**Published:** 2026-02-18

**Authors:** Lennart Bedarf, Kathrin Gerlach, Holger Wittig, Eva Scheurer, Celine Berger, Claudia Lenz

**Affiliations:** 1 Institute of Forensic Medicine, Department of Biomedical Engineering, University of Basel, Basel, Switzerland; 2 Institute of Forensic Medicine, Health Department Basel-Stadt, Basel, Switzerland; 3 Institute of Forensic Medicine, Pestalozzistrasse 22, Basel, 4056 Switzerland

**Keywords:** Postmortem interval, Forehead temperature, Time of death, Henssge, Postmortem body cooling

## Abstract

Estimating the time of death (ToD) and correspondingly the postmortem interval (PMI) is a fundamental aspect of forensic medicine, essential for event reconstruction and legal investigations. Traditional methods based on the body core temperature can be influenced by external and physiological factors, potentially leading to uncertainties. This study explores a non-invasive approach using forehead temperature measurements. It offers several advantages over assessing the body core temperature, including reduced sensitivity to clothing insulation and fat composition of the deceased, making it a promising alternative for forensic applications. Temperature data from 31 deceased individuals were analysed using a double-exponential fitting function, achieving high accuracy with a root mean square error of 0.25 °C and a coefficient of determination (R²) of 0.999, indicating a robust model. Compared to traditional methods, the approach presented in this study helps to minimize the influence of individual body factors, although it may still be affected by environmental factors such as sunlight exposure, varying ambient temperature and airflow. The method performs best for shorter PMIs, likely due to the forehead’s faster cooling compared to the body core. Further research is required to validate its performance under varied conditions and to assess its comparability with established approaches.

## Introduction

Estimating the time of death (ToD) and the postmortem interval (PMI) is critical in forensic medicine, playing a key role in event reconstruction and legal investigations. Accurate determination of ToD provides valuable insights for forensic pathologists, law enforcement, and judicial authorities. Over the years, various methods have been developed for the forensic routine estimation of ToD, primarily to narrow down the time window of death, including:

- Algor mortis [1–5];
- Livor mortis and rigor mortis analysis [6–13];
- Supravital reactions [14–17]
- Biochemical markers [18];
- Forensic entomology [19]
- Decomposition rate studies [20, 21].

Henssge et al. developed methods for estimating the time of death based on core body temperatures, including the brain and the rectal temperatures [3, 4]. To date, the Henssge nomogram based on the rectal temperature is the gold standard for estimating ToD. The accuracy of ToD estimation using the rectal temperature is affected by various factors that influence the body’s cooling rate such as the environmental temperature, clothing, body composition, environmental humidity, rate of autolysis, air movement, and the starting temperature at ToD [1–3, 22, 23]. To account for these factors, Henssge introduced corrective factors; however, these can only be approximated and do not fully overcome the limitations associated with them. Moreover, obtaining accurate body weight at the scene is often impractical, further complicating reliability. Therefore, alternative, non-invasive methods, which are less influenced by external factors are highly desirable in forensic medicine.

One technique uses finite element models to simulate the heat flow [24]. Moreover, another existing framework is the so-called Prism method, which is based on the mathematical description by Marshall and Hoare [25–27] in combination with corrective factors introduced by Henssge [28]. Besides those procedures, the use of temperature measurements in the head region gained rising attention. It is possible to use the brain temperature, as well as the temperature profile of the external auditory canal or the eye temperature [29–31]. The forehead region appears promising due to its unique thermal dissipation properties. Unlike body core temperature measurements, the forehead temperature cooling is less affected by clothing or fat layers of the deceased, potentially offering a more reliable indicator of postmortem temperature changes [32]. Moreover, in contrast to rectal temperature measurements, forehead temperature measurements are less susceptible to inter-operator variability and less prone to procedural complications [33]. Furthermore, forehead temperature measurements offer the advantage of being non-invasive and have been demonstrated to correlate closely with brain temperature [32]. Moreover, Henssge and others have described brain temperature as a reliable parameter for estimating the time of death. In contrast, assessing the rectal temperature might carry potential risks, including bladder or rectal perforation [34–38].

This study presents a theoretical framework, as well as the development of a novel method for ToD estimation based on the forehead temperature. A parametric fitting function is proposed to model the decline in forehead temperature over time. By utilizing the unique thermal properties of the forehead, this approach aims to lay a foundation for improved practicability in the ToD estimation in forensic investigations.

## Materials and methods

**Fig. 1:**
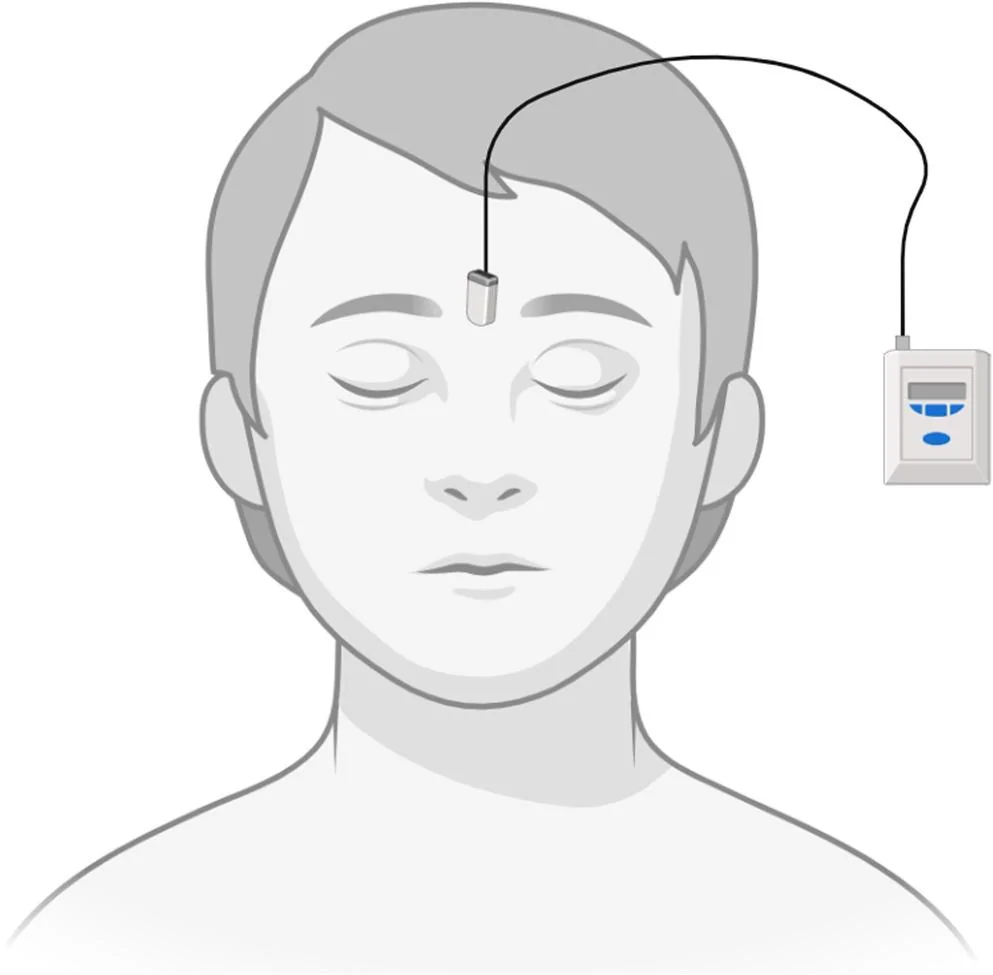
Illustration of the temperature probe positioned 2 cm above the glabella, centred between the eyebrows

Forehead temperature measurements were conducted on 31 deceased individuals (18 females, 13 males, age 66 ± 13 years). The deceased have been included in the study because of their precisely known ToD. The ToD was either provided by assisted suicide organizations or marked by the termination of resuscitation efforts by emergency medical services. Of the 31 cases examined, 30 involved assisted suicide involving the administration of pentobarbital sodium. The remaining one’s cause of death was attributed to acute right heart failure. All cases involved deaths that occurred indoors. After death, each body underwent a legal inspection at the place of death, which was performed by forensic pathologists who also completed the death certificate. As part of the forensic routine procedure, a temperature probe was placed on the forehead 2 cm above the glabella, centred between the eyebrows, as shown in Fig. 1. Forehead and environmental temperature measurements were recorded by a portable data logger system (175 T2, Testo SE & Co., Mönchaltorf, Switzerland). Prior to transportation, the bodies remained at the location of death under environmental temperatures of 20–22 °C. During the following hours (mean 4 h 29 min ± 18 min), the deceased were transported to the cooling chamber (2.5 °C) of the local Institute of Forensic Medicine. Transportation time was less than 45 min for all investigated cases. The temperature probe continuously collected temperature measurements every 10 s until the measured forehead temperature arrived at the equilibrium temperature of approximately 2.5 °C inside the cooling chamber.

Following data collection, further processing of the temperature curves from all subjects was performed in MATLAB 2024b (The MathWorks, Inc., Natick, MA, United States) [39]. To ensure comparability, all individual temperature curves were aligned to the exact ToD, provided from the certificate of death. The mean cooling curve was calculated for the entire period after death, including both pre-cooling and cooling phases. The following fitting function based on a double-exponential model, as described by Marshall and Hoare, as well as Henssge [40–43] was applied to the mean cooling curve:

1 $$\:\frac{T\left(t\right)-{T}_{a}}{{T}_{0}-{T}_{a}}=\alpha\:{e}^{-\beta\:t}-\gamma\:{e}^{-\delta\:t}$$

with T: temperature, t: time, T_a_: ambient temperature, T_0_: starting temperature.

To reduce the risk of overfitting, only one data point every two minutes was included in the fitting procedure. The parameters α, β, γ, δ, as well as their corresponding standard deviations, were derived during the curve fitting process. T_0_ was set to 33.3 °C. To encompass the full physiological variation of forehead surface temperatures observed in living individuals, two additional boundary conditions were introduced, using T_0_ = 31.0 °C and T_0_ = 35.6 °C. These limits reflect the empirically established range of 31.0–35.6 °C reported by Ng et al. [44].

The resulting fit parameters enabled the estimation of the ToD by incorporating the measured forehead temperature, the ambient temperature at the time of examination, and the fitted temperature decay function [Eq. 1].

## Results

**Fig. 2:**
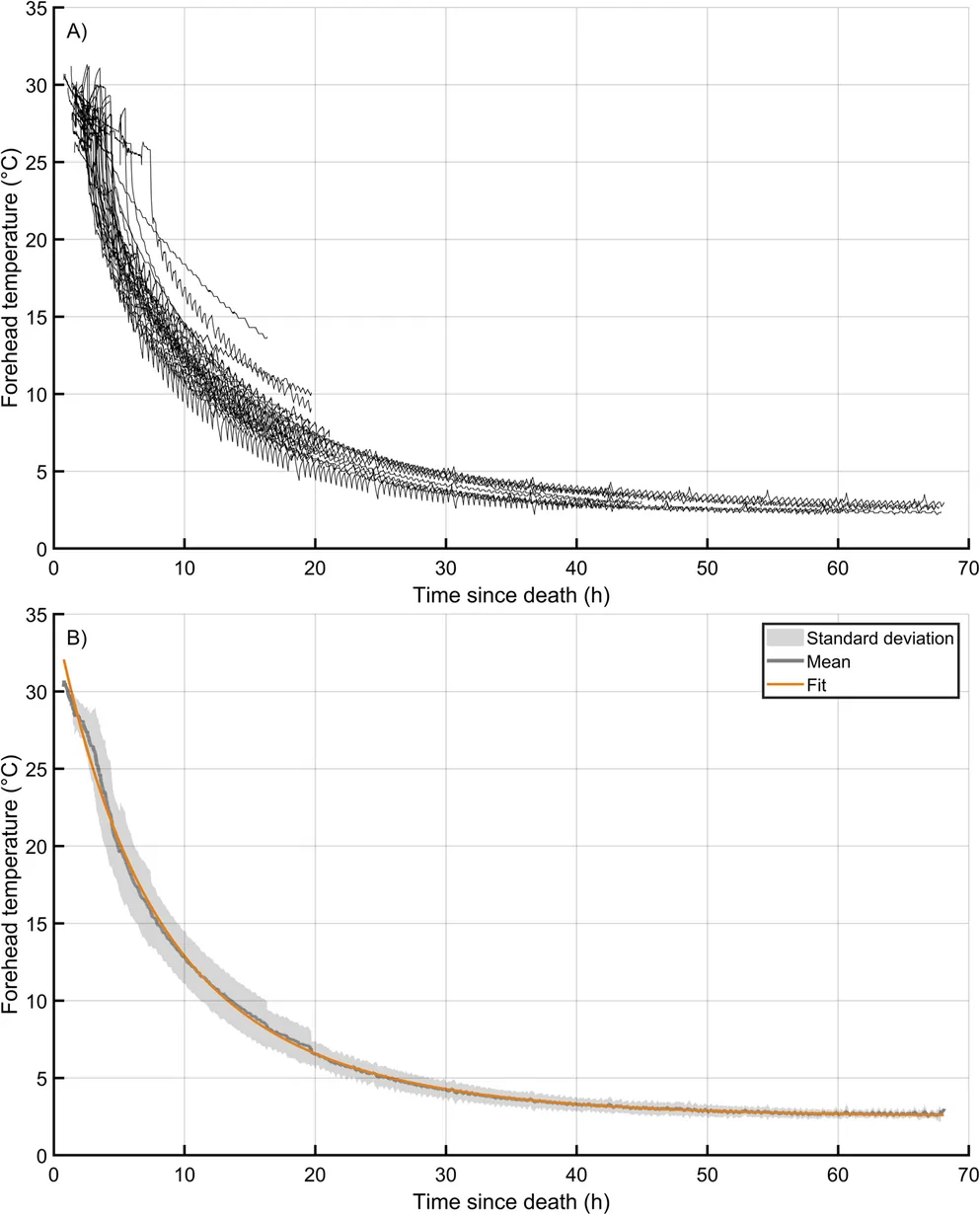
(**A**) Individually acquired temperature measurements as a function of the time since death. (**B**) Averaged forehead temperature across all subjects (grey line) including the corresponding standard deviation (shaded grey area), and the calculated fit function (orange)

Figure 2 illustrates the individual temperature measurements, as well as the average temperature curve, corresponding standard deviation, and the fitted curve. The difference between the temperature of a living person and the start of the measurements arises due to the delay between ToD and the finished legal inspection. Table 1 summarizes the resulting fit parameters.

The summarized fit model can therefore be written as:[Eq. 2].

2 $$\:T\left(t\right)=\left(0.510\cdot\:{e}^{-0.183\:\cdot\:t}-\left(-0.548\right)\cdot\:{e}^{-0.076\:\cdot\:t}\right)\cdot\left({T}_{0}-{T}_{a}\right)+{T}_{a}$$

**Fig. 3:**
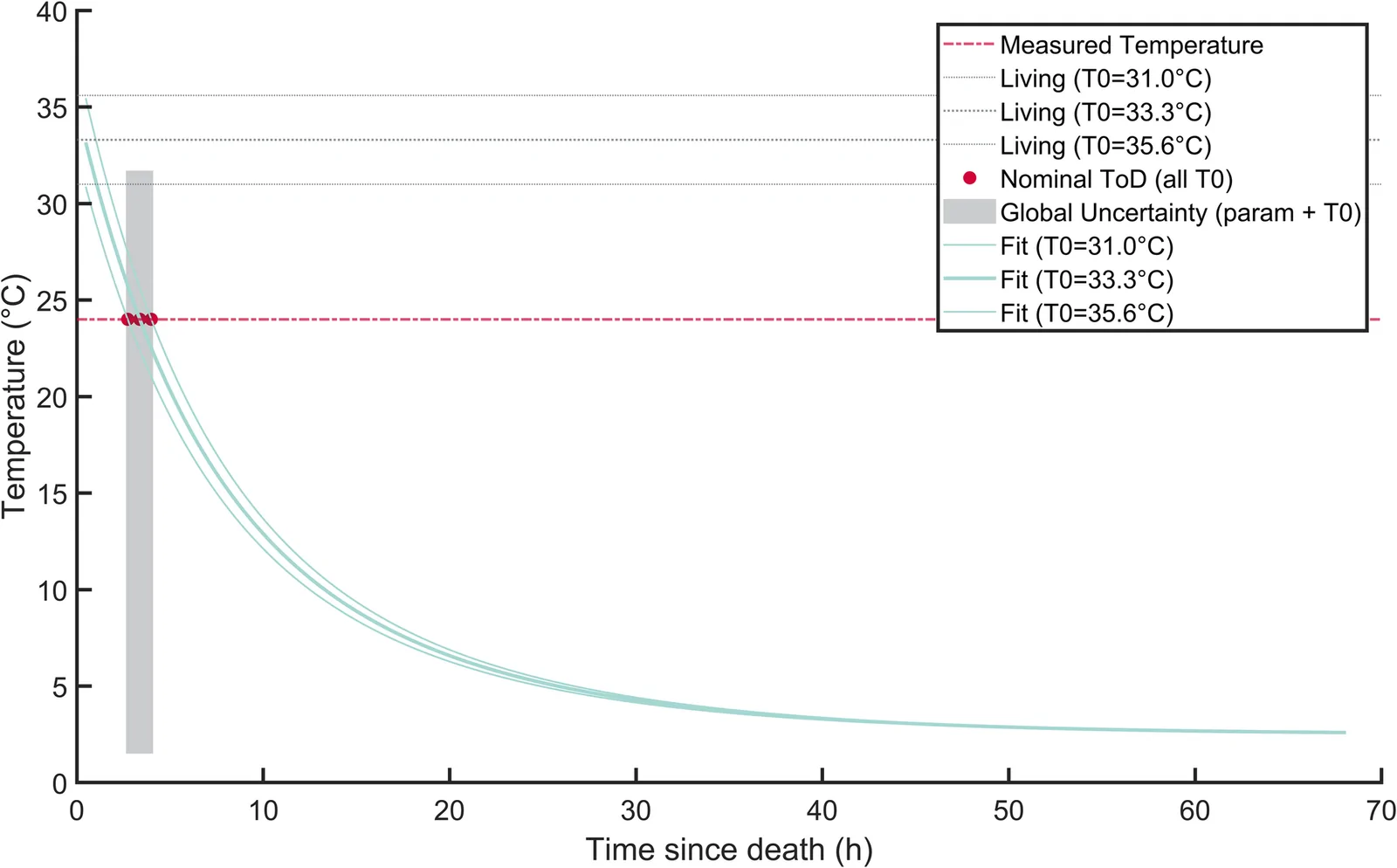
Graphical depiction of the estimated ToD based on an exemplary forehead temperature of 24 °C and an ambient temperature of 2.5 °C. The uncertainty range is indicated by the grey area going through the estimated ToDs

The fit model achieved a root mean square error (RMSE) of 0.25 °C and a coefficient of determination (R^2^) of 0.999. As illustrated in Fig. 3, the model estimates a postmortem interval of 3.38 h (3.29 –3.48 h) for an exemplary forehead temperature of 24 °C and an exemplary ambient temperature of 2.5 °C, when calculating with the mean T_0_ of 33.3 °C. In addition, the ToD was calculated for two standard deviations ranging from T_0_ = 31 °C to T_0_ = 35.6 °C covering 95% of all forehead temperatures detected in the empirical study by Ng et al. [44]. Due to these factors the calculated range of possible ToDs rises to 2.64 –4.09 h. In this case, we also calculated the 95% confidence interval for the initial forehead temperature of 33.3 °C. The interval ranged from 3.378 h to 3.386 h, which is considerably smaller than the variation caused by natural changes in T_0_.

**Table 1:** Resulting fit parameters and corresponding standard deviations

$\:\alpha\:$	$\:\beta\:$	$\:\gamma\:$	$\:\delta\:$
$\:0.5101\pm\:0.00520$	$\:0.1829\pm\:0.0013$	$\:-0.5477\pm\:0.0055$	$\:0.0763\pm\:0.0003$

## Discussion

Traditional methods for estimating ToD, as for example the widely used Henssge nomogram, are based on the measurement of core body temperatures such as the rectal and brain temperature [3, 4]. However, these approaches are subject to several limitations, including the practical and technical challenges of the temperature measurements. The body core temperatures are further limited by influencing of factors including covering of the body and body composition [1–5, 22, 23]. Previous work showed that the forehead surface temperature can reliably model the temperature behaviour of the brain [32]. Building on these findings, the present study aimed to overcome the limitations associated with traditional core thermometries by developing a novel model for ToD estimation based on postmortem forehead surface temperature measurements.

The observed forehead’s cooling profiles show only minor inter-individual variability and appear to be largely unaffected by differences in body variations or fat composition [2]. Notably, there is no visible temperature plateau following the time of death (ToD) or subsequent placement in the cooling chamber - an important advantage over core body temperature measurements [45]. This phenomenon can be attributed to the fact that the body surface is in direct contact with the ambient environment and undergoes immediate postmortem cooling. As a result, no postmortem temperature plateau is observed externally, which is typically associated with internal body temperature measurements, where initial heat retention delays the onset of cooling [32, 46]. Additionally, the forehead is positioned directly over the frontal bone of the skull, which is known to have a high thermal conductivity compared to fatty tissues [47, 48]. Due to this fact our fitting approach does not include an additional parameter for the compensation of a temperature plateau. The fitting parameters naturally reflect the continuous decline observed in the recorded forehead temperatures. Unlike areas where the body composition can vary significantly, the forehead has relatively consistent anatomical characteristics across individuals of different body sizes [49, 50]. Besides less influence from individual variations in body composition, forehead temperature measurements also demonstrate fewer procedural complications compared to rectal temperature measurements, thereby enhancing its reliability and practical applicability. However, it should be noted that the data in our study were obtained under controlled conditions. All cases involved deaths that occurred indoors, and the bodies were placed in the cooling chamber within a few hours after death. Therefore, this approach is not yet generalizable but represents an initial investigation providing a foundation for future studies under varying environmental conditions.

Our fitting results demonstrate that the double-exponential cooling model according to Henssge [Eq. 1] accurately describes the postmortem decline of the forehead temperature at the given conditions [51]. With the parameters (𝛼, 𝛽, 𝛾 and 𝛿) derived from empirical data, the model achieves high accuracy, as evidenced by the resulting RMSE of 0.25 °C and R² of 0.999. The low standard deviation of the parameters suggest that the model can provide consistent results when applied to cases with comparable scenarios.

While the forehead appears to be a convenient and reliable site for temperature measurements, its surface temperature might still be influenced by environmental factors such as direct sunlight or drafts. In contrast, the rectal temperature is less susceptible to environmental fluctuations due to the relatively larger mass and volume of the torso compared to the head, which provides greater thermal insulation from ambient conditions. Furthermore, the reduced insulation of the head leads to reduced cooling time of the forehead in comparison to the body’s core temperature, thereby shortening the window for ToD estimation.

This study’s findings are based on postmortem experiments performed under rigorously controlled conditions, reflecting the standardized environmental parameters specified by Henssge [3]. Hence, the application of the ToD estimation using the forehead temperature is restricted to controlled conditions. Future studies will be required to investigate the applicability of the ToD estimation using the forehead temperature under differing environmental conditions.

## Conclusion

This study evaluates the forehead temperature for the estimation of the PMI. The derived model achieved exceptional performance, with an RMSE of 0.25 °C and an R² of 0.999, demonstrating its high robustness and precision at the given conditions. Compared to traditional body core temperature methods, the forehead-based approach is less affected by inter-individual anatomical and physiological variability. However, more research is needed to validate this approach across diverse environmental conditions before it can be implemented in forensic practice.
